# [^18^F]DPA-714-PET-MRI reveals pronounced innate immunity in human anti-LGI1 and anti-CASPR2 limbic encephalitis

**DOI:** 10.1007/s00415-024-12302-7

**Published:** 2024-03-12

**Authors:** Wolfgang Roll, Jan Bauer, Andre Dik, Christoph Mueller, Philipp Backhaus, Saskia Räuber, Bastian Zinnhardt, Marco Gallus, Catriona Wimberley, Peter Körtvelyessy, Philipp Schindler, Werner Stenzel, Christian E. Elger, Albert Becker, Jan Lewerenz, Heinz Wiendl, Sven G. Meuth, Michael Schäfers, Nico Melzer

**Affiliations:** 1 Department of Nuclear Medicine, University of Münster, Münster, Germany; 2 Department of Neuroimmunology, Center for Brain Research, Medical University of Vienna, Vienna, Austria; 3 Department of Neurology with Institute of Translational Neurology, University of Münster, Münster, Germany; 4 European Institute for Molecular Imaging, University of Münster, Münster, Germany; 5 Department of Neurology, Medical Faculty and University Hospital, Heinrich Heine University of Düsseldorf, Moorenstraße 5, 40225 Düsseldorf, Germany; 6 Biomarkers and Translational Technologies (BTT), Pharma Research & Early Development (pRED), F.Hoffmann-La Roche Ltd., Basel, Switzerland; 7 Department of Neurological Surgery, University of California San Francisco, San Francisco, USA; 8 Edinburgh Imaging, University of Edinburgh, Edinburgh, UK; 9 Department of Neurology, Campus Benjamin Franklin, Charité-Universitätsmedizin Berlin, Corporate Member of Freie Universität Berlin and Humboldt-Universität zu Berlin, Berlin, Germany; 10 Department of Clinical Radiology, University of Münster, Münster, Germany; 11 Department of Neuropathology, and Berlin Institute of Health (BIH), Charité-Universitätsmedizin Berlin, Corporate Member of Freie Universität Berlin, Humboldt-Universität zu Berlin, Berlin, Germany; 12 Department of Epileptology, University of Bonn, Bonn, Germany; 13 Section for Translational Epilepsy Research, Department of Neuropathology, University of Bonn, Bonn, Germany; 14 Department of Neurology, University of Ulm, Ulm, Germany

## Dear Sirs,

A major cause of mesial temporal lobe seizures and epilepsy, memory disturbance and psychiatric symptoms is autoimmune limbic encephalitis (ALE), mediated by adaptive and concomitant innate autoimmune inflammatory processes. Standard clinical workup comprises magnetic resonance imaging (MRI), electroencephalography (EEG), CSF analysis, and neuropsychological assessment [1].

ALE with autoantibodies (AABs), against leucine-rich, glioma inactivated (LGI1) and contactin-associated protein-like 2 (CASPR2) belong to the most frequent subtypes of ALE. In these ALE entities, MRI and EEG often display unspecific and very subtle changes, routine CSF analysis is often unremarkable [2, 3], and no specific pattern of cognitive dysfunction exists [4]. Even AAB testing using cell-based assays may yield false-positive and -negative results [5]. Hence, novel biomarkers directly addressing the parenchymal immune response are urgently warranted to enhance diagnostic accuracy together with AAB testing.

[^18^F]DPA-714 is a second-generation PET-tracer targeting the 18 kDa translocator-protein (TSPO), overexpressed on the mitochondrial membrane of activated microglia and other innate immune cells [6, 7]. Previous studies provided data on increased TSPO expression, suggesting ongoing inflammation, in mesial temporal seizure foci and in contralateral mesial temporal lobe [8] with similar inflammatory changes found in brain tissue specimen of ALE [9].

Here, we aimed at corroborating the potential of TSPO-PET-MRI as a novel diagnostic imaging marker for the assessment of innate immunity in human ALE with AABs against LGI1 and CASPR2 given the fact that antigen-bound AABs have been shown to yield a microglia response in the brain parenchyma [10]. A focus of this work was on the immunohistochemical crossvalidation of the TSPO-PET signal on the cellular level.

Two ALE patients underwent combined [^18^F]DPA-714-PET-MRI as compassionate use. Patients underwent routine clinical evaluation in the University Hospital of Münster, Germany. Diagnosis of ALE was based on current consensus criteria [1, 4]. Retrospective analysis was approved by local ethics committee (Ethikkommission der Ärztekammer Westfalen-Lippe; reference number 2013–350-f-S and 2021–144-f-S, and from the Medical University of Vienna, EK 1206/2013) and was performed in accordance with the principles of the 1964 Declaration of Helsinki and its later amendments. All patients gave written informed consent.

Neuropsychological assessments were performed after recovery from seizures. Verbal memory scores were extracted for left temporal lobe cognitive function and visual memory scores for right temporal lobe, as described previously [4].

Standard 10–20 surface electrode systems with additional anterior temporal electrodes for short-term- and basal temporal electrodes for long-term-EEG were used. The EEG records were rated regarding interictal epileptic discharges/slowing or ictal events confined to anterior temporal electrodes in an unilateral or bilateral fashion as previously described [4].

Cell counts, protein and immunoglobulin levels as well as the presence of IgG AAB against intracellular and neural surface membrane antigens in serum and CSF were analyzed as previously described [4]. Viral, fungal and bacterial pathogens, and rheumatological-vasculitic disorders were ruled out.

[^18^F]DPA-714 was prepared automatically in a GE TRACERlab MX module as described in detail previously [7].

Hybrid Imaging was performed on a 3 T PET-MRI (mMR; Siemens Healthcare). Dynamic PET was acquired in list mode after injection of 237/258 MBq [^18^F]DPA-714 for 60 min after injection. MRI included non-contrast enhanced sequences: isotropic (1 mm) 3D structural T1-weighted, axial T2-weighted-sequences and axial/coronar FLAIR.

3D T1-weighted MR images were processed with Freesurfer (http://surfer.nmr.mgh.harvard.edu/), as previously described [11]. Relative volume of hippocampus and amygdala to intracerebral volume were used for further analysis.

After coregistration with segmented T1-weighted MR images, regional standardized uptake value ratio (SUVR) of the [^18^F]DPA-714 PET were extracted, using the cerebellar grey matter as reference region [11].

Patients’ blood samples were analyzed for single nucleotide polymorphism c.439A > G (rs6971, p.Thr147Ala), known to affect the binding affinity of TSPO-PET-tracers, as described previously [7].

Sections from control (Autopsy brain from male, 71 years without neurological disease) and two different ALE patients with identical AAB (LGI1, CASPR2) were stained for TSPO as previously described using anti-TSPO (Abcam, ab109497, 1:1.000) antibodies [11].

Multiplex immunofluorescent labeling was performed with antibodies against TSPO (Abcam, ab109497, 1:10.000), neurons (NeuN; Merck MAB377, 1:2500), oligodendrocytes [12] (TPPP/p25 (1:5000, kind gift from Romana Höftberger), astrocytes (GFAP, Thermo Scient. #MS-1376, 1:1000), and microglia/macrophages (Iba-1, Wako #019–19741, 1:10.000) by utilizing the Akoya Fluorescent Multiplex kit according to the manufacturer’s protocol [11].

Two patients with AABs against surface membrane neural antigens underwent [^18^F]DPA-714-PET-MRI. Both patients were high affinity binders without TSPO polymorphism. Both patients received high-dose corticosteroid therapy (patient #1: 500 mg/d for 5 days; patient #2: 1000 mg/d for 3 days) followed by tapering and immunoabsorption therapy (5 cycles). Afterwards patients received an induction therapy with 1000 mg Rituximab. Patient #1 received additional maintenance therapy with 1000 mg Rituximab 6 months and 1 year after initial diagnosis. Patient #2 received an additional dose of 1000 mg Rituximab 2 weeks after the first cycle, followed by 4 more cycles of Rituximab during the following 2 years.

A 71-year-old patient with seropositive ALE (patient #1) was hospitalized following the occurrence of memory deficits and impulsiveness. LGI1 AABs were detected in serum (titer 1:100) but not in CSF. CSF routine analysis results were as follows: cell count 0/µl; glucose: 68.4 mg/dl, oligoclonal bands: type 5; IgG Index: <0.7.

**Fig. 1:**
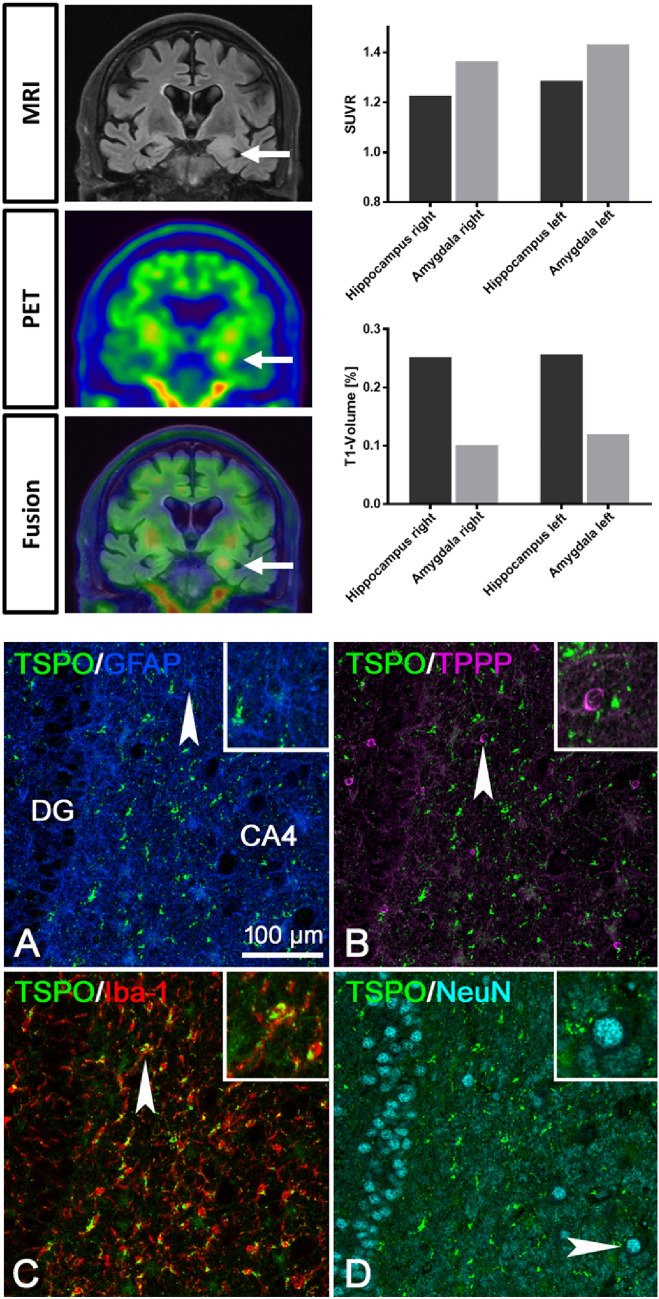
FLAIR-MRI, [18F]DPA-714 PET and fused images (upper left) of patient #1 with anti-LGI1 autoimmune limbic encephalitis. Quantification of the SUVR and relative T1 volumes of amygdala and hippocampus of patient #1 (upper right). Multiplex staining, in brain tissue samples of an independent patient with anti-LGI1 autoimmune limbic encephalitis obtained from epilepsy surgery for seizure control, for TSPO together with GFAP (**A**), TPPP/p25 (**B**), Iba-1 (**C**) and NeuN (**D**) in the hippocampus. Strong TSPO mitochondrial reactivity is only seen in the Iba-1^+^ microglial cells. GFAP^+^ astrocytes and TPPP/p25^+^ oligodendrocytes show much weaker TSPO reactivity. No double labeling for TSPO is seen in NeuN^+^ neurons. The insets in A-D show higher magnifications of the single cells indicated by the arrowheads

[^18^F]DPA-714-PET-MRI (Fig. 1) before initiation of immunotherapy showed asymmetrically elevated tracer uptake with punctum maximum in the left amygdala (SUVR; left: 1.431; right: 1.364) and hippocampus (SUVR; left: 1.287; right: 1.227) compared to the contralateral hemisphere (Fig. 1). Uptake was above cerebellar gray matter, used as reference region for the calculation of SUVR. Asymmetrical uptake correlated with FLAIR signal alterations with temporomesial edema in the left hemisphere. Consistently EEG revealed anterior temporal sharp-slow-waves and slowing on the left hemisphere. No significant mesial temporal cognitive dissociation with asymmetrical mesial temporal dysfunction was found (z-score left: – 2.28; right: – 2.32).

Relative T1 volume of amygdala (left: 0.120; right: 0.101) and hippocampus (left: 0.257; right: 0.252) did not show relevant lateralization. One year after TSPO-PET patient #1 reported a subjectively complete recovery with normalization of the volume/signal increase of the left temporomesial region in T2/FLAIR-MRI and non-detectable serum AAB against LGI1.

A 65-year-old patient with seropositive ALE (patient #2) and AABs against CASPR2 in serum (titer 1:3200) and CSF (titer 1:320) was hospitalized following recurrent temporal lobe seizures and associated memory deficits. CSF routine analysis results were as follows: cell count 6/µl; glucose: 61.3 mg/dl, oligoclonal bands: type 1; IgG Index: <0.7.

**Fig. 2:**
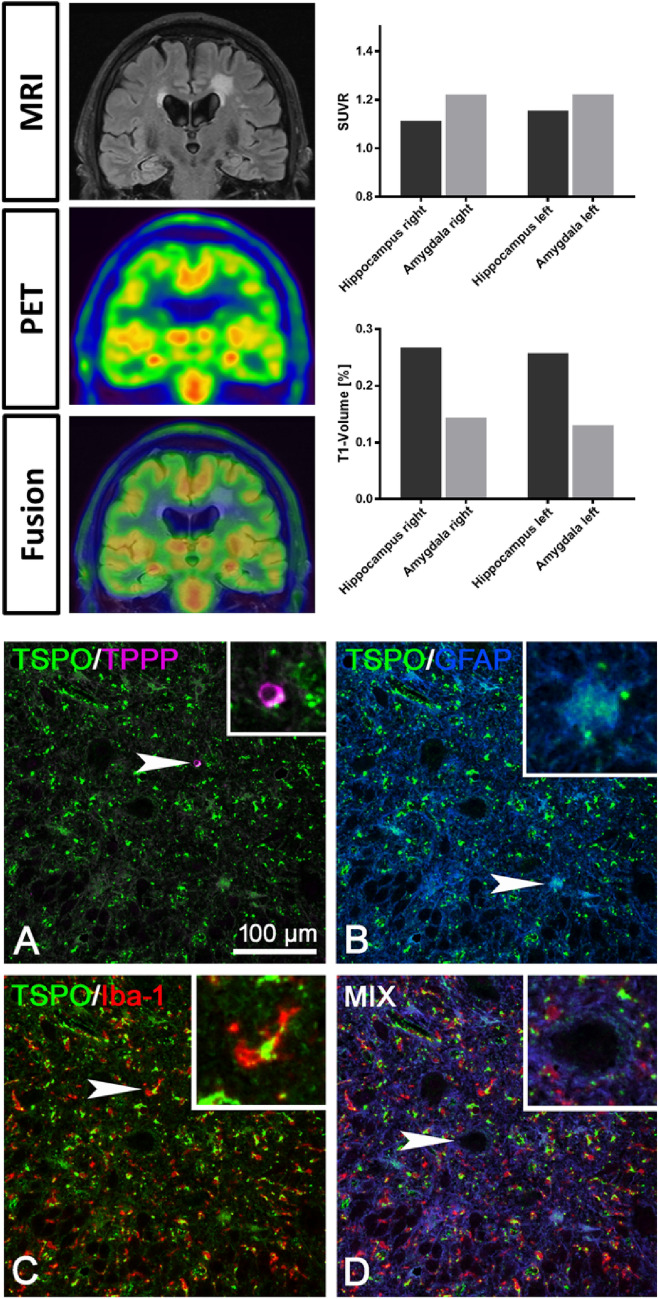
FLAIR-MRI, [18F]DPA-714 PET and fused images (upper left) of patient #2 with anti-CASPR2 autoimmune limbic encephalitis. Quantification of the SUVR and relative T1 volumes of amygdala and hippocampus of patient #2 (upper right). Multiplex staining, in brain tissue samples of an independent patient with anti-CASPR2 autoimmune limbic encephalitis, obtained from epilepsy surgery for seizure control, for TSPO together with TPPP/p25 (**A**), GFAP (**B**), Iba-1 (**C**) and quadruple staining for TSPO, TPPP/p25, GFAP and Iba-1 (**D**). In addition, here, strong TSPO mitochondrial reactivity is only seen in Iba-1^+^ microglial cells. GFAP^+^ astrocytes and TPPP/p25^+^ oligodendrocytes show much weaker TSPO reactivity. The arrowhead (enlarged in the inset) here points at a large neuron that is negative for TSPO. The insets in A-D show higher magnifications of the single cells indicated by the arrowheads

In [^18^F]DPA-714-PET-MRI (Fig. 2) before initiation of immunotherapy quantitative uptake values were above cerebellar reference region for amygdala (SUVR; left: 1.222; right: 1.222) and hippocampus (SUVR; left: 1.155; right: 1.114), however, not asymmetrical as in patient #1. FLAIR images revealed nearly symmetrical signal alterations in mesial temporal lobes of both hemispheres. Consistently in EEG, abnormalities occurred in both hemispheres with anterior temporal slowing. Neuropsychological testing showed cognitive dissociation with dominant dysfunction in visual memory in comparison to verbal memory, indicating right mesial temporal impairment (z-score left: -0.27; right: -2.12) in contrast to symmetrical alterations in [^18^F]DPA-714-PET, FLAIR and EEG.

Relative T1 volume of amygdala (left: 0.130; right: 0.144) and hippocampus (left: 0.258; right: 0.268) again did not show relevant lateralization.

Two years after TSPO-PET, patient #2 had a normalization of previously observed EEG changes and of the volume/signal increase of the temporomesial region in T2/FLAIR-MRI. CASPR2 AABs remained positive.

**Fig. 3:**
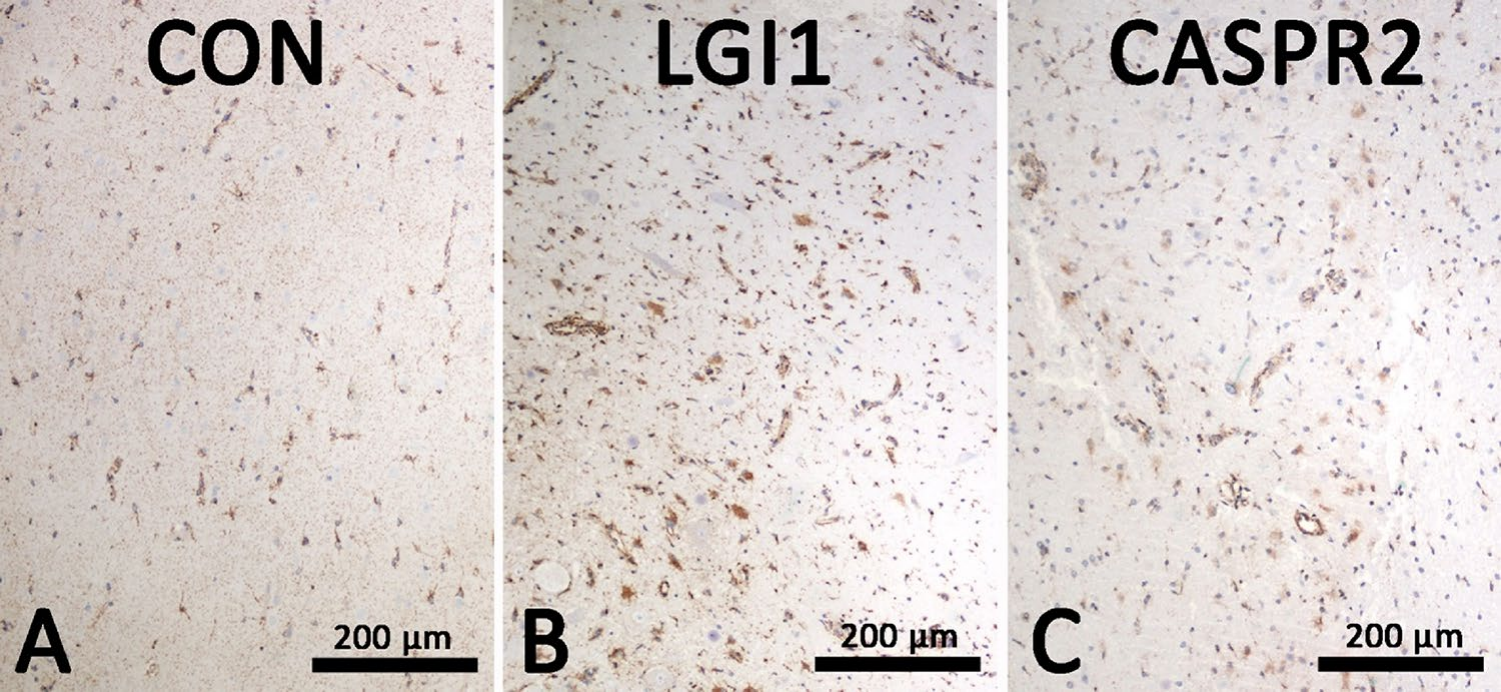
TSPO staining was performed in **A** control brain (71 year with no neurological disease), **B** anti-LGI1 autoimmune limbic encephalitis brain and **C** anti-CASPR2 autoimmune limbic encephalitis brain. Whereas in control brain, a moderate expression of TSPO in all glial cells is seen, in anti-LGI1 and anti-CASPR2 autoimmune limbic encephalitis brain, activated glial cells show an increased reactivity for TSPO. Neurons in control as well as LGI1 and CASPR encephalitis brain are negative for TSPO

In control brain, moderate expression of TSPO was equally detected in oligodendrocytes, astrocytes and microglial cells. Neuronal cell bodies were negative (Fig. 3). In both anti-LGI1 (Fig. 1C) and anti-CASPR2 (Fig. 2C) ALE brain (staining for TSPO was stronger in activated glial cells although neurons remained negative (Figs. 1D and 2D). Multiplex immunofluorescence imaging showed that in anti-LGI1 ALE brain (Fig. 1) especially the Iba-1 + microglial cells showed strong expression of TSPO. Here, GFAP + astrocytes and TPPP/p25 + oligodendrocytes showed weaker expression of TSPO. NeuN + neurons showed absence of TSPO reactivity. A comparable TSPO reactivity was seen in the anti-CASPR2 ALE case (Fig. 2) with strong TSPO expression in microglial cells, less intense reactivity in astrocytes and oligodendrocytes and absence of TSPO expression in neurons.

Our data from two ALE patients with AABs against surface membrane neural antigens are in line with previously published results showing elevated temporomesial [^18^F]DPA-714 uptake in patients with temporal lobe epilepsy in a bilateral symmetric or asymmetric fashion suggesting ongoing inflammation inside and outside of current seizure foci [8]. Our findings are further consistent with the notion that ALE is a bilateral albeit often asymmetric disease [1]. In accordance with findings in seronegative and seropositive ALE with AABs against intracellular neural antigens, the [^18^F]DPA-714 signal correlated with FLAIR-MRI and EEG alterations but not with neuropsychological assessments and T1-volumetry [11]. In this study, clear evidence argued for FLAIR signal increase and EEG abnormalities being down-stream effects of an antibody-mediated neural effector mechanism [13, 14]. Consistently, it has recently been demonstrated that antigen-bound parenchymal AABs via Fc receptor signaling also elicit such a parenchymal microglia response [10]. Although AABs in anti-LGI1 and anti-CASPR2 ALE are often not detectable in CSF [2, 3], they can be retrieved as monoclonal AABs from intrathecal antibody secreting B-cell populations and exert functional effects consistent with their pathogenic parenchymal effect [15].

Indeed, imaging TSPO expression in infiltrating and parenchymal immune cells allows for the detection of Iba-1 + phagocytes as the main source not only in ALE, but also in the myeloid tumor microenvironment [6], and cerebral vasculitis [7]. GFAP + astrocytes contribute to the TSPO-PET signal to a lower amount [6, 7]. Moreover, in the previous studies, the [^18^F]DPA-714-PET signal exceeded the MRI abnormalities [7]. Thus, [^18^F]DPA-714-PET-MRI might allow for the imaging of key pathological processes in inflammatory CNS diseases. Larger studies have to define the role of [^18^F]DPA-714-PET compared to standard MRI in the clinical setting. It is important to address not only imaging at initial diagnosis, but also for response assessment during/after immunotherapy. Questions arising on the specificity of the signal over the time of the disease should be addressed in dedicated preclinical models, in comparison to changes in FLAIR/T2 signal alterations.

Limitations of our study include method inherent disadvantages of [^18^F]DPA-714-PET-MRI discussed in previous publications [6, 7]. First to mention is limited availability of PET-MR systems and tracers as [^18^F]DPA-714 [8, 11]. Small patient number and matched PET-MRI and histopathological specimen from different patients are a further limitation. A major limitation of [^18^F]DPA-714 is reliable quantification and specificity of tracer binding. Gold standard for the assessment of specific binding to the target is kinetic modeling out of dynamic PET-datasets. In a previous analysis, we were able to show that binding potentials calculated by kinetic modeling showed a very high correlation to SUVR with cerebellar grey matter as reference region used in this study [11]. Comparison to healthy controls would further strengthen our results. However, preclinical evaluation of [^18^F]DPA-714-PET-MRI in an experimental setup with histopathological correlation underlined specificity of the PET signal in human ALE [11]. Potential spillover from the choroid plexus is another disadvantage of [^18^F]DPA-714-PET, but can be limited when using automatic brain segmentation as in this study [8, 11]. Neuropsychological assessment is hampered by the fact that nonverbal memory performance is associated with both temporal lobes [4]. Future studies should include 3D FLAIR allowing for reliable volumetry in a larger patient cohort.

To conclude, we provide preliminary data on the potential [^18^F]DPA-714-PET-MRI as a direct imaging maker of neuroinflammation in ALE with antibodies against surface membrane neural antigens. Larger studies are needed to define the abilities of [^18^F]DPA-714-PET-MRI for clinical and treatment monitoring purposes in comparison to standard of care.

## Data Availability

All data generated or analyzed during the current study are included in this published article.
